# The Fascial Breath

**DOI:** 10.7759/cureus.5208

**Published:** 2019-07-23

**Authors:** Bruno Bordoni, Marta Simonelli, Bruno Morabito

**Affiliations:** 1 Cardiology, Foundation Don Carlo Gnocchi, Milan, ITA; 2 Osteopathy, French-Italian School of Osteopathy, Pisa, ITA; 3 Osteopathy, School of Osteopathic Centre for Research and Studies, Milan, ITA

**Keywords:** fascia, myofascial, diaphragm, breathing, osteopathic, physiotherapy

## Abstract

The word diaphragm comes from the Greek (διάϕραγμα), which meant something that divides, but also expressed a concept related to emotions and intellect. Breath is part of a concept of symmorphosis, that is the maximum ability to adapt to multiple functional questions in a defined biological context. The act of breathing determines and defines our holobiont: how we react and who we are. The article reviews the fascial structure that involves and forms the diaphragm muscle with the aim of changing the vision of this complex muscle: from an anatomical and mechanistic form to a fractal and asynchronous form. Another step forward for understanding the diaphragm muscle is that it is not only covered, penetrated and made up of connective tissue, but the contractile tissue itself is a fascial tissue with the same embryological derivation. All the diaphragm muscle is fascia.

## Introduction and background

The body is rich in liquids such as blood and lymph. The cell is rich in liquid as well as between cells and tissues. What keeps liquids together and what makes them circulate and communicate? The fascia. How to define the fascia? The discovery of new functions and features of the fascia makes the definitions like the same colour under a different light: changing [1-5]. In a recent work by our research group, Foundation of Osteopathic Research and Clinical Endorsement (FORCE), we defined the fascia as: “The fascia is any tissue that contains features capable of responding to mechanical stimuli. The fascial continuum is the result of the evolution of the perfect synergy among different tissues, liquids and solids, capable of supporting, dividing, penetrating, feeding and connecting all the districts of the body, from the epidermis to the bone, involving all the functions and organic structures. The continuum constantly transmits and receives mechanometabolic information that can influence the shape and function of the entire body. These afferent/efferent impulses come from the fascia and the tissues that are not considered as part of the fascia in a biunivocal mode. In this definition, these tissues are included: epidermis, dermis, fat, blood, lymph, blood and lymphatic vessels, tissue covering the nervous filaments (endoneurium, perineurium, epineurium), voluntary striated muscle fibers and the tissue covering and permeating it (epimysium, perimysium, endomysium), ligaments, tendons, aponeurosis, cartilage, bones, meninges, tongue” [6]. The article briefly reviews the concept of fascia and the fascial structure that involves and forms the diaphragm muscle with the aim of changing the vision of this complex muscle: from an anatomical and mechanistic form to a sentient and homothety form.

## Review

Fascial tissue

**Figure 1:**
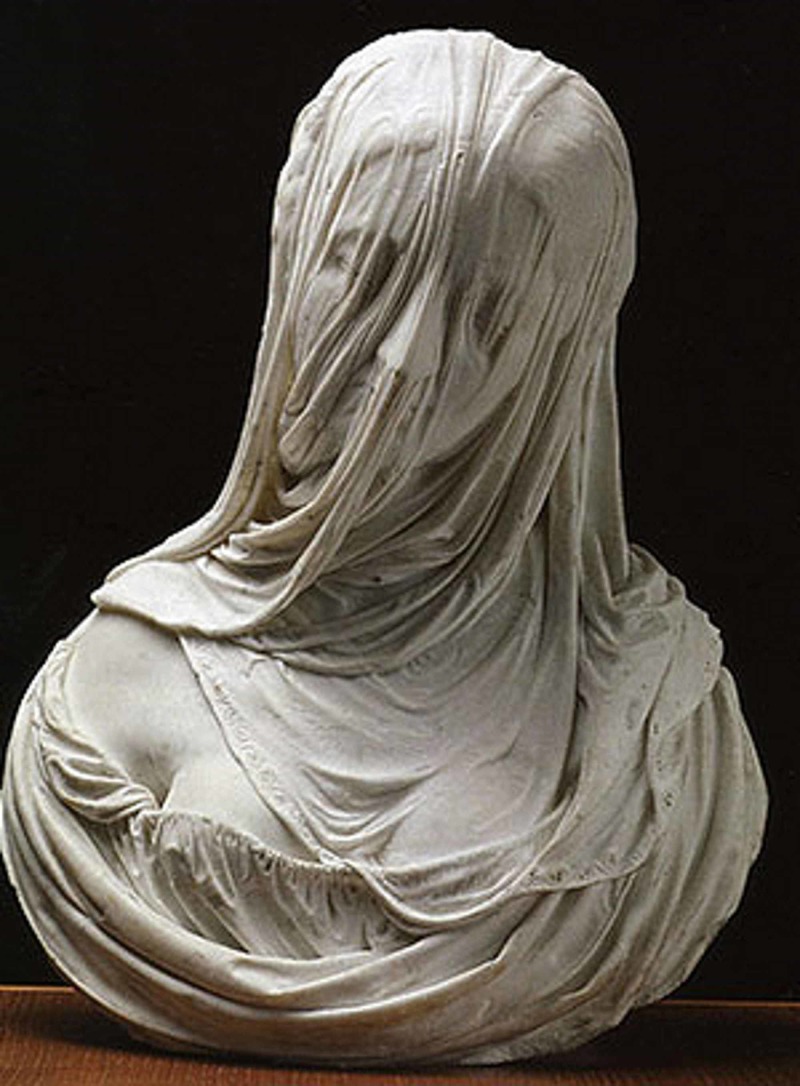
The statue of the “Busto di Donna Velata” by the sculptor Corradini (1717)

The fascia is not just connective tissue but is a more complex and vital structure. In the image below, the statue of the “Busto di Donna Velata” by the sculptor Corradini (1717), it is highlighted that the fascia covers and constitutes what we are, but it also influences our emotional expression (Figure 1).

Not only does the fascia influence the expression of the solid part of the human body such as the transmission of force through the myofascial system, the maintenance of the organs in its own seat, the posture, but can influence the emotional status [7-10]. The fascial continuum has a multitude of receptors: myelinated proprioceptive terminations of the myofascial system (Ruffini, Golgi and Pacini); free endings without myelin sheath in contact with the periosteum, the connective tissue of all viscera and the connective tissue of the striated muscles [10]. All receptors involving the fascial continuum are appointed to the functions of proprioception, nociception and interoception [10]. The interoceptive routes project information to the medullary centres and to the brainstem, where they are sorted to the anterior cingulate cortex and the posterior dorsal insula, thanks to the thalamus-cortical extension. The afferents of endomysium and perimysium without myelin sheath are related to myelinated afferent neurons (type III or alpha-delta fibres), and non-myelinated (type IV or fiber-C). C-fibres can activate the brain areas involved in emotional expression (insular cortex); the mechanical deformation to which a muscle is subjected stimulates these afferents, which reach the insula [10]. The fascia can influence health status, altering the mechanometabolic environment, causing clinical pictures of pain, inflammation and possible tumour formation [11-17]. A chronic deformation of the myofascial system causes the mechanoreceptors to turn into nociceptors, for example in the thoracolumbar fascia, simulating an idiopathic back pain syndrome [15]. A decline in the sliding of fascial tissues causes a picture of local inflammation, as shown for some types of cervicalgia [18]. An altered position of the collagen fibres, for example in a tendon, could change its mechanical function, creating pain but not inflammation [19]. If the layers and fascial orientations of an anatomical area lose the ability to move between them, the vectors of collagen fibrils will change, there will be an implementation of collagen deposition, creating a metabolic environment of inflammation and anomalous mechanical tension of the scaffold cell and extracellular matrix. This fibrosis or desmoplasia is one of the stimuli to create and maintain a tumour phenomenon. According to a current of thought, the excess production of transforming growth factor-beta (TGFβ) from fibrotic tissue is stimulated, which will increase the production of collagen and fibrosis: a vicious circle for the invasion of tumour cells [17]. Maintaining an optimal position of the collagen fibres, adapted to a specific anatomical structure (muscle, joint, visceral capsule, meninges, etc.), means maintaining health [5,19-20]. The fascial tissue has memory and awareness: "We are not dealing only with a tissue, but with awareness." [21]. The deformation of the cell is an immediate strategy to know the external environment and allow the structures that make up the same cell to adapt, according to the principle of mechanotransduction. During cellular deformation, ribonucleic acid (RNA) interference (RNAi) and deoxyribonucleic acid (DNA) are involved, which are fundamental for the learning of the cell, the memory of what happened and for the transport of information outside the cell to other tissues [21-22]. In this way, each cell of the fascial continuum can communicate with distant tissues. In the mechanism of mechanotransduction, the cytoskeleton plays a major role, thanks to a metabolic regulator [target of rapamycin (TOR)] which plays an important role in cellular morphological memory [21]. TOR influences actin polymerization, which in turn collects information outside the cell through ramifications that push against the cytoskeleton, forming small ripples (lamellipodium) or more pronounced deformations (filopodium) [21]. These phenomena are transient. At the end of actin, inside the cell, resides the myosin that pulls in the opposite direction to the expansion of actin; in this way, mechanical tension is created which exits and enters the cell. The journey of mechanotransduction information is in microseconds, with information that reaches the cell genes almost instantaneously [21]. Other structures that support the cellular capacity to perceive what happens outside the cell, to influence the morphology and retain the memory of mechanotransduction events are microtubules (MTs) or microtubule-associated proteins (MAPs). These proteins transport vibrations (determined by morphological variations) and electromagnetic information (created by the same vibrations) towards the DNA and towards other cells. MAPs can be compared to a cell's own nervous system, as the transport of such information is rapid and has an influence on the behaviour of other cells and tissues that spread like wildfire: "This mechanism can be compared to a conscious awareness " [21]. The fascial tissue has memory, in order to adapt better and awareness, that is, the ability to prepare the cells in the presence of a stressor (internal or external), through varied and extremely rapid means of communication [21]. In the solid tissue considered as a fascia, fibroblasts and telocytes can create branches to put more cells in contact simultaneously and for considerable distances. The gap junctions exist between fibroblasts, which are made up of two proteins (connexons, consisting of homomeric or heteromeric connexins) [23]. These gap junctions allow the transport of mechanical, metabolic and electrical information. Telocytes can expand their cellular processes into very long filaments (telopodes), very thin (podomeres) or thicker and dilated (podoms) [24]. These telopodes can come into contact with other telocytes, fibroblasts and other cells at inside the fascial continuum. Telocytes play essential roles in mechanical, metabolic, cellular and immune repair processes [24]. The fascial tissue is a network interconnected with other networks (collagen, cells, cytoskeleton, protein filaments) and immersed in liquids (blood and lymph, extracellular matrix, cellular liquids). When we think of fascial tissue, we should not imagine a network but a "wetwork". The fascial tissue is compared to a biotensegretive complex, a term coming from a concept of architecture (tensegrity) [1]. Comparing the fascial continuum to a tensegretive structure when we do not yet have scientific elements to describe this concept in the presence of liquids, is like talking about the sky night without the stars: useless. Perhaps we should talk about the tensegrity of liquids in a solid context: fascintegrity. The diaphragm muscle falls within the fascial continuum. Breath is part of a concept of symmetry, that is, maximum capacity adaptive to multiple functional questions in a defined biological context [25]. The act of breathing determines and defines our holobiont: how we react and who we are.

The connective tissue of the diaphragm muscle

The diaphragmatic fascia is made up of the layers that cover and divide the septa and the muscle fibres (epimysium, perimysium, endomysium), as well as the attack of the different muscular parts on the vertebrae and ribs. The medial pillars will affect the dorsal vertebrae (D11-D12) and lumbar vertebrae (up to maximum L4), leaning on the vertebral periosteum, through a connective thickening [26-27]. The intermediate pillars merge with their own epimysium and with the epimysium of the body of the diaphragm muscle and with the epimysium of the intermediate pillars. The lateral pillars attach themselves to the twelfth rib with the epimysium and merge with the epimysium of the large psoas and quadratus lumborum muscle [26-27]. The muscular body of the diaphragm merges with the epimysium at the endothoracic costal fascia, which last covers the entire upper portion of the diaphragm [12]. The lower portion of the diaphragm is covered by the transversalis fascia, continuation of the endothoracic fascia [12]. The posterior diaphragmatic area is covered by the thoracolumbar fascia [13,15]. The central portion of the diaphragm or central tendon muscle or phrenic centre is pure connective tissue [26]. The extracellular matrix is rich in collagen fibrils, as well as the basement membrane [1,28]. Another step forward for understanding the diaphragm muscle is that it is not only covered, penetrated and made up of connective tissue but also the contractile tissue itself is fascial tissue [1-2]. All the diaphragm muscle is fascia. The connective tissue and contractile tissue derive from the same embryological leaflet, the mesoderm [3]. The diaphragm muscle is a stratification of multiple fascial networks: transversalis fascia, endothoracic fascia, thoracolumbar fascia, phrenic centre, epimysium, perimysium, endomysium, basement membrane, extracellular matrix, contractile tissue. Each network must cooperate, allowing the diaphragmatic complex to contract and relax during the breaths or to allow the diaphragm to perform other tasks such as postural tasks or to allow the passage of the food bolus [13]. The diaphragm is not a plunger, it does not contract and release uniformly, but with different times and methods depending on the task to which it is called to work [28-29]. The connective networks are organized in an entropic manner, so as to allow the muscular complex to act with different vectors and support multiple stressors [5]. We move from a Bernstein model of movement (recognizable and mechanistic patterns) to a fractal model of muscle contraction (one recognizes the three-dimensionality of the construct in an environment that is continually transformed) [28,30-31]. Muscle efficiency depends on a fractal and non-mechanistic system [30]. One should imagine the movement of the diaphragm as a homothety (mathematical and geometric term), that is, a continuous change of form and function while maintaining its identity, and with asynchronous contractile timing [32-33]. The diaphragm, like any fascial tissue, has the memory of its mechanical behaviour (morphological alteration of the different structures that make up the muscle) and possesses awareness [21]. The fascia has the ability to anticipate a morphological alteration, recordable through different vital parameters (heartbeat and skin conductance) before the stressful event occurs [21]. Probably, this system of awareness reflects the electromagnetic fields that every cell and tissue produce. The magnetic variations deform the cells, which stimulates the cellular function and the adaptation of the mechanometabolic environment, up to the DNA. An electromagnetic field travels faster than the electric conduction and can go beyond the body barrier [21]. A fascial system can affect the fascial system of another individual. Two muscles of different individuals can synchronize their electromyographic response during a movement [34]. One of the possible explanations for this phenomenon is the presence of electromagnetic fields produced by the fascial system. Another explanation for this fascial awareness can be linked to the nervous system capable of anticipating a phenomenon [35]. The tissues making up the diaphragm could be predisposed to an emotional or mechanical event thanks to the central nervous system, to protect itself by the stressful event (longer or shorter breath, body movements). This ability to adapt before a mechanical event could be a strategy not yet understood and studied, that our body uses for maximum efficiency and survival.

## Conclusions

The diaphragm muscle is fascial tissue, including the connective tissue (epimysium, perimysium, endomysium, tendons, phrenic centre) and the contractile portion. The diaphragmatic movement is complex and is influenced by the surrounding environment and by the organization of its tissues. Muscle contraction and relaxation are fractal and asynchronous. Muscle tissues can prepare for a mechanical event before the event is present, as a kind of awareness. The most likely explanation is related to the action of the central nervous system and to electromagnetic fields produced by the cells of the human body. We are still far from understanding in detail the behaviour of this fascinating muscle.
